# Improved Pathogenicity of a Beet Black Scorch Virus Variant by Low Temperature and Co-infection with Its Satellite RNA

**DOI:** 10.3389/fmicb.2016.01771

**Published:** 2016-11-04

**Authors:** Jin Xu, Deshui Liu, Yongliang Zhang, Ying Wang, Chenggui Han, Dawei Li, Jia-Lin Yu, Xian-Bing Wang

**Affiliations:** ^1^State Key Laboratory of Agro-Biotechnology, China Agricultural UniversityBeijing, China; ^2^Key Laboratory of Pollinating Insect Biology, Ministry of Agriculture, Institute of Apicultural Research, Chinese Academy of Agricultural SciencesBeijing, China

**Keywords:** beet black scorch virus, satellite RNA, RNA silencing, temperature, siRNAs

## Abstract

Co-infection of none-coding satellite RNAs (sat-RNAs) usually inhibits replication and attenuates disease symptoms of helper viruses. However, we find that the sat-RNA of *Beet black scorch virus* (BBSV) and low temperature (18°C) additively enhance the systemic infection of BBSV in *Nicotiana benthamiana*. Northern blotting hybridization revealed a relatively reduced accumulation of BBSV-derived small interfering RNAs (siRNAs) in presence of sat-RNA as compared to that of BBSV alone. Cloning and sequencing of total small RNAs showed that more than 50% of the total small RNAs sequenced from BBSV-infected plants were BBSV-siRNAs, whereas the abundance of sat-siRNAs were higher than BBSV-siRNAs in the sat-RNA co-infected plants, indicating that the sat-RNA occupies most of the silencing components and possibly relieves the RNA silencing-mediated defense against BBSV. Interestingly, the 5′ termini of siRNAs derived from BBSV and sat-RNA were dominated by Uridines (U) and Adenines (A), respectively. Besides, the infection of BBSV alone and with sat-RNA induce down-regulation of miR168 and miR403, respectively, which leads to high accumulation of their targets, *Argonaute 1* (*AGO1*) and *AGO2*. Our work reveals the profiles of siRNAs of BBSV and sat-RNA and provides an additional clue to investigate the complicated interaction between the helper virus and sat-RNA.

## Introduction

The satellite RNAs (sat-RNAs) of plant viruses rely on the helper virus for replication and encapsidation but share little or no sequence similarity with the helper virus genome (Murant and Mayo, 1982; Hu et al., 2009). Based on genome size, the sat-RNAs are divided into two classes: large sat-RNAs encoding a nonstructural protein and small sat-RNAs without coding capacity (Simon et al., 2004). Sat-RNAs usually inhibit the replication of helper viruses and may alter the disease symptoms of the helper viruses depending on the interaction among host factors, helper viruses, and sat-RNAs (Collmer and Howell, 1992; Roossinck et al., 1992; Hull, 2002). Despite the fact that there are sat-RNAs that reduce the disease symptoms without influencing the accumulation of helper viruses, most sat-RNAs reduce both disease symptoms and titer of the helper virus, probably by competing with the helper virus for common replication factors (Roossinck et al., 1992; Hull, 2002). However, there are exceptions in which sat-RNAs enhance the disease symptoms during co-infections. For example, a few isolates of Cucumber mosaic virus (CMV) sat-RNAs can exacerbate the disease symptoms in tobacco and tomato hosts because of a specific sequence carried by the sat-RNAs (review in Roossinck et al., 1992; Simon et al., 2004). Similarly, sat-RNA of *Groundnut rosette virus* (GRV) is largely responsible for the groundnut rosette symptom and the transmission between natural hosts of GRV (Murant et al., 1988; Robinson et al., 1999). Interestingly, unlike the sat-RNAs of CMV and GRV, the sat-RNA C of *Turnip crinkle virus* (TCV) often enhances the visible symptoms of TCV in a host-dependent manner (Li, 1990). Since sat-RNA C is capable of interfering the encapsidation of TCV genomic RNAs thereby to boost the accumulation of free coat proteins, which are RNA silencing suppressor (Wang and Simon, 1999; Thomas et al., 2003; Zhang and Simon, 2003), these observations suggest that some sat-RNAs enhance helper virus disease symptoms by directly or indirectly suppressing RNA silencing. So far, to our knowledge, there is no report to document an enhancement of helper virus titers during co-infection with sat-RNAs (Hull, 2002; Simon et al., 2004).

In the past few years, the progressive understanding of RNA silencing results in more information about the interaction between plants and the pathogens. The RNA silencing in plants has been found to have numerous functions in the course of plant growth (Baulcombe, 2004; Meister and Tuschl, 2004; Chapman and Carrington, 2007), among which one of the important function is to provide an adaptive immune system counteracting pathogens, including viruses, bacteria, and so on (Ding et al., 2004; Voinnet, 2005; Ding and Voinnet, 2007). In *Arabidopsis*, the cascade of DCL2/3/4, AGO1/2/3/5/7/10, and RDR1/6 have been shown to be involved in antiviral RNA silencing pathways (review in Huang et al., 2016). Accordingly, the viruses have evolved diverse viral suppressors of RNA silencing (VSRs) to inhibit distinct steps in the silencing pathway (Li and Ding, 2006; Díaz-Pendón and Ding, 2008). At present, about 35 individual VSR families have been reported in plant viruses (Ding and Voinnet, 2007). The co-evolution processes of silencing and suppressing reveal complex interaction between virus and host plant in the long history of co-existence (Ding and Voinnet, 2007). Some subviral pathogens, such as viroids and satellites, are also influenced by the pressure of RNA silencing and evolved effective secondary structures to avoid or minimize the small RNA-mediated silencing (Wang et al., 2004). The secondary structure of *Potato spindle-tube viroid* (PSTVd) was also found to induce silencing but could be resistant to RISC-mediated cleavage (Itaya et al., 2007). Recent studies demonstrate that sat-RNAs-derived siRNAs can directly silence host genes, which is responsible for sat-RNA-induced disease symptom (Shimura et al., 2011; Smith et al., 2011).

*Beet black scorch virus* (BBSV) was firstly reported in northern China in the late 1980s and lately identified as a new species of genus *Betanecrovirus* (Cao et al., 2002; Yuan et al., 2006; King et al., 2011). BBSV induced the symptom of black scorched leaves and necrotic fibrous roots in the sugar beet plants in late spring, causing severe yield loss in the plantation areas. Two isolates from the provinces of Ningxia and Xinjiang have been reported in China, designated as BBSV-N and BBSV-X respectively (Cao et al., 2002; Xi et al., 2006), which exhibit 99.45% similarity in nucleotide sequence. In addition, BBSV-Co was reported in Colorado of Unite States (Weiland et al., 2006, 2007), which shared 93% similarity with BBSV-N, and different BBSV isolates were identified in Iran and Spain (Koenig and Valizadeh, 2008; González-Vázquez et al., 2009). During the serial propagation of BBSV, a gain-of-function mutant harboring a single nucleotide substitution at nucleotide (nt) 3477 in the 3′UTR induce higher infectivity than wild-type BBSV in *N. benthamiana* (Xu et al., 2012). In addition to the viral genome RNA, another 615 nt single stranded RNA has been identified as a satellite RNA in the isolate of BBSV-X (Guo et al., 2005). During the replication of sat-RNA of BBSV, various forms such as monomers, dimers, and tetramers are accumulated, and the dimer form plays an intermediate role in replication (Guo et al., 2005).

In this study, we first showed that the satellite RNA enhance the pathogenesis and accumulation of BBSV in *N. benthamiana* plants under at or below room temperature. Further analyses including cloning and sequencing of siRNAs derived from BBSV and its sat-RNA, suggest that sat-RNA may alleviate RNAi mediated antiviral silencing to enhance the systemic infection of BBSV by acting as surrogacy of the helper virus.

## Materials and methods

### Plant materials and virus inoculation

*N. benthamiana* plants were grown in a growth chamber with a 16-h-light/8-h-dark cycle at 25°C. Three leaves of *N. benthamiana*, typically at the four-leaf stage in 1-month-old, were used for inoculation (Xu et al., 2012). A BBSV variant, BBSV-m294 (abbreviated as Bm, GenBank accession no. JN635330.1) that caused severe symptom in *N. benthamiana* and obtained after a passage of propagation (Xu et al., 2012). The sat-RNA (GenBank accession no. NC_006460.1) of BBSV were used for inoculation by the method reported previously (Xu et al., 2012). After inoculation, plants were grown at 18 and 25°C conditions. Three systemic leaves were harvested at 12 dpi for northern blot analysis and sequencing of small RNA.

### RNA isolation and northern blot analysis

Total RNA was extracted using TRIzol® Reagent (Invitrogen, USA). For detection of viral genomic RNA and mRNA of BBSV and its sat-RNA, 2 μg total RNA extracted from mock or virus-infected plants was used for hybridization using indicated gene-specific ^32^P-radiolabled cDNA probes corresponding to the 3′UTR fragment of BBSV or the full-length sat-RNA, respectively as described (Xu et al., 2012). For small RNA gel blots, 10 μg total RNA were separated on 17% denaturing polyacrylamide gel (PAGE) and transferred to nylon membranes (GE Healthcare, UK). DNA oligonucleotides corresponding to the sequences of BBSV (nt 155–176, nt 769–788, nt 823–842, nt 1259–1278, nt 1762–1781, nt 2020–2039, nt 2266–2287, and nt 3115–3134) or sat-RNA (nt 132–152, nt 395–416 and nt 506–527) were synthesized respectively. The mixtures of antisense oligonucleotides corresponding to BBSV and sat-RNA were labeled with [γ-^32^P] ATP as probes used for hybridization at 40°C for 16–20 h in PerfectHyb plus buffer (Sigma-Aldrich). The membranes were washed in 2 × SSC (0.3 M NaCl and 0.03 M sodium citrate) containing 0.2% SDS for 30 min and then twice with 1 × SSC containing 0.1% SDS for 20 min both at 50°C.

### Small RNA library sequencing and analysis

The small RNA libraries were generated following the manufacturer's protocol (Illumina, California, USA). Briefly, separated by electrophoresis, RNA fractions with sizes between 18–30 nt corresponding to the small RNA population were purified, and cloned using NEBNext® Multiplex Small RNA Library Prep Set for Illumina kit. The final products were quantified on the Agilent DNA 1000 chip and sequencing was performed by use of an Illumina Hiseq 2500-SE50 (Illumina, California, USA). The Illumina sequencing reads were first trimmed to remove the adaptor sequence to get clean reads. The trimmed sequencing reads were then blasted to the Bm (JN635330.1) and sat-RNA (NC_006460.1) and the sequences with full matched were considered as Bm or sat-RNA small RNAs. The clean reads were also blasted in miRBase for miRNAs. The data of small RNA libraries was deposited in GenBank with accession number GSE80694.

### Quantitative RT-PCR analyses

To measure expression levels of miR168 and miR403, stem-loop quantitative RT-PCR technique was used as previously described (Varkonyi-Gasic and Hellens, 2011). The quantitative expression of DCLs, AGOs and RDR6 mRNAs were checked by real-time RT-PCR as described previously (Kotakis et al., 2010). Primers used for quantitative analysis above are listed (Table S1).

## Results

### Low temperature or sat-RNA co-infection enhances the infection by BBSV variant m294

**Figure 1:**
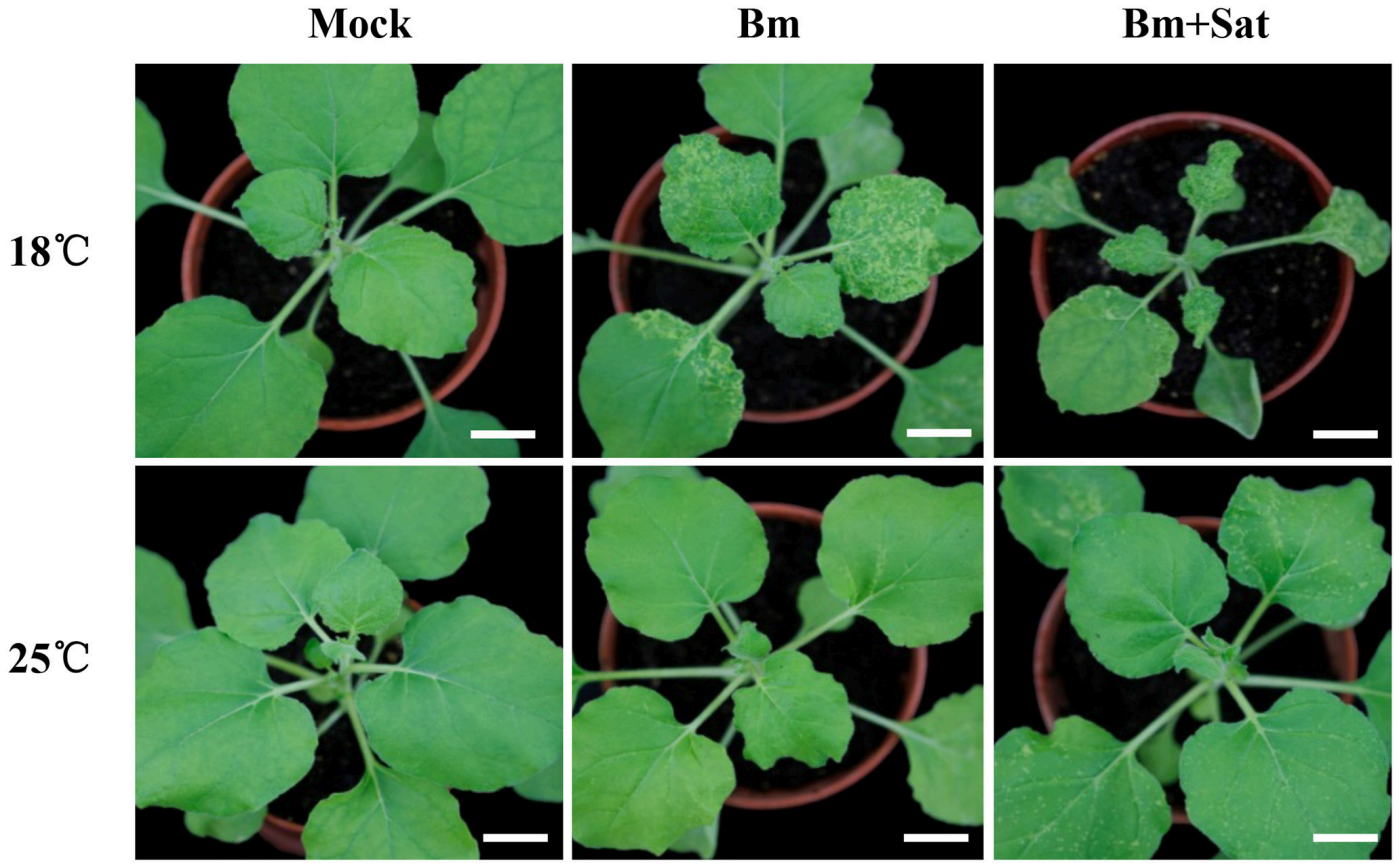
**Co-infection of sat-RNA and low temperature additively improved the systemic infectivity of BBSV**. Symptom development in *N. benthamiana* inoculated by buffer (Mock), Bm alone and co-infected with its sat-RNA at 18 and 25°C. The infected seedlings were photographed at 12 days after inoculation with purified Bm or sat-RNA-associated Bm at a concentration of 100 μg/mL. Bars = 2 cm.

In our previous studies, a series of BBSV spontaneous variants were isolated from serial propagation of wild-type BBSV in *Chenopodium amaranticolor* and *N. benthamiana*. The typical variant BBSV-m294 (abbreviated as Bm, GenBank accession no. JN635330.1) causes more severe symptoms than wild-type BBSV at low temperature (18°C) (Xu et al., 2012). To determine the impact of environment temperature on Bm infection, *N. benthamiana* plants were mechanically inoculated with Bm and maintained at 18 or 25°C, respectively. At 12 dpi, the infected plants were photographed as shown in Figure 1. The Bm-infected plants induced typical yellow curling symptoms on systemic leaves at 18°C, whereas the Bm induced very few yellow chlorotic mottle spots in upper leaves at 25°C (Figure 1, middle panel).

BBSV infection is naturally associated with satellite RNAs (sat-RNAs), which depend on BBSV for replication and movement but share no sequence homology with the helper viral genome (Guo et al., 2005). To determine if the sat-RNA affects the pathogenicity of BBSV in different temperatures, we further inoculated *N. benthamiana* plants with Bm alone or with its sat-RNA at 18 or 25°C. In contrast with very few infection lesions by Bm alone, clearly visible disease symptom was observed in the systemically infected leaves infected by Bm and its sat-RNA at 25°C (Figure 1, bottom panel). Moreover, the viral symptom induced by Bm and its sat-RNA was further enhanced at 18°C than at 25°C (Figure 1, upper panel).

Collectively, both low temperature and co-infection with sat-RNA additively enhance the pathogenicity of BBSV.

### Sat-RNA co-infection enhances Bm accumulation but reduces the production of Bm-derived siRNAs

**Figure 2:**
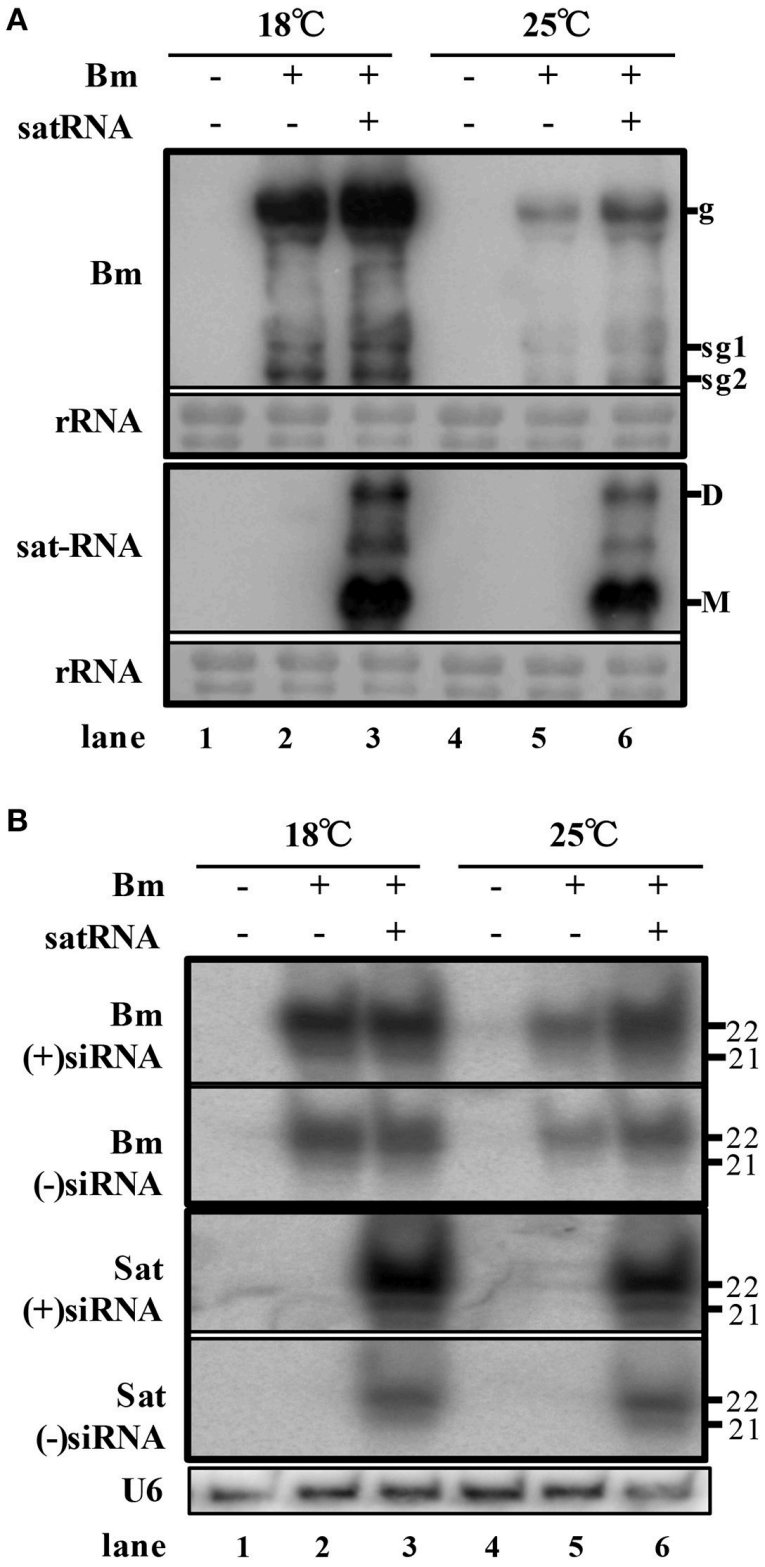
**Low temperature and co-infection with its sat-RNA relatively relieve the silencing potency targeting to BBSV**. **(A)** Accumulation of viral RNA from Bm (top panel) and sat-RNA (bottom panel) in systemically infected leaves. Genomic (g) and sub-genomic RNA1 and RNA2 (sg1 and sg2) were indicated on the right at the top panel. Sat-RNA monomer (M) and dimer (D) were indicated on the right at the bottom panel. Methylene blue-stained rRNA was used as a loading control. **(B)** northern blot analysis of Bm- and sat-RNA -derived siRNAs by probes hybridizing to sense and anti-sense genome regions. U6 were indicated as loading controls. The positions of 21 and 22 nt RNAs are indicated on the right.

In order to examine the accumulation of BBSV along with or without its sat-RNA in different temperature, we further carried out northern blot hybridizations to detect the genomic and subgenomic RNA of Bm, as well as sat-RNA. In consistence with symptom observations, the genomic and subgenomic RNA of Bm accumulated to significantly higher levels at 18°C than that of Bm at 25°C (Figure 2A, lane 2 and 5). In addition, the accumulation level of Bm genomic RNA was higher in sat-RNA co-infection samples than Bm alone at either low or room temperature (Figure 2A, upper panel). Thus, these results indicate that both low temperature and co-infection with its sat-RNA additively enhance Bm accumulation in *N. benthamiana* plants.

To investigate the RNA silencing-mediated antiviral defense, the accumulation of the small interfering RNAs derived from the sat-RNA and its helper virus were analyzed through northern blot hybridizations. The Bm- and sat-RNA-derived siRNAs were readily detected in systemic leaves and all the viral siRNAs were mostly 22-nt in length followed by 21-nt (Figure 2B), which revealed that the replications of Bm and its sat-RNA strongly triggered the host RNA silencing. The accumulation level of BBSV-derived siRNAs was similar in the plants infected by Bm alone and co-infection with sat-RNA at 18°C (Figure 2B, compare lane 2 and 3), despite of the fact that BBSV genomic RNA accumulated to higher levels in the presence of sat-RNA than that of Bm alone (Figure 2A, compare lane 2 and 3). This finding indicated that the presence of sat-RNA relatively decreased the production of Bm-derived siRNAs in the co-infected plants at 18°C. Significantly, sat-siRNAs accumulated to very high levels in the co-infected leaves, regardless of temperature conditions (Figure 2B, sat-siRNAs lane 3 and 6). These results demonstrated that the high-level accumulation of the sat-RNA and its derived siRNAs may saturate the potency of antiviral silencing targeting Bm, which relieves the silencing targeting to Bm.

### Sat-RNA reduces the production of Bm-derived siRNAs by saturating DCL2 and DCL4 function during co-infection

To characterize the population of the siRNAs derived from Bm and its sat-RNA, total small RNAs were cloned from the systemically infected leaves of *N. benthamiana* plants maintained at different temperatures. After trimming the linker sequences, the small RNA reads of 18- to 30-nt in length were further analyzed. More than 16 million small RNA reads, including endogenous small RNAs and virus-derived siRNAs, were obtained from each sample (Table 1). Notably, in the Bm-infected leaves, approximately 69.2% of total sequenced small RNAs were perfectly match or complementary to the genome of Bm (Table 1), indicating that Bm genomic and subgenomic RNAs served as the major substrates of the host Dicer enzyme(s) in *N. benthamiana* plants infected with Bm alone. In the BBSV/sat-RNA co-infected plants at 18°C, however, 29.6 and 43.2% of total small RNAs were mapped to Bm and its sat-RNA, respectively (Table 1). The similar results were obtained in the systemic leaves infected by Bm alone or with sat-RNA at 25°C (Table 1). These results are consistent with the above northern blot analysis and strongly indicate that sat-RNA is the predominant substrate of the host Dicer enzyme(s), leading to reduced production ratio of the helper viral siRNAs in the BBSV/sat-RNA co-infected plants.

**Table 1:** **The amount of small RNAs isolated from systemic leaves of ***N. benthamiana*** inoculated with buffer (Mock), Bm alone or co-inoculated with the sat-RNA at low (18°C) and room (25°C) temperature**.

	**18°C Mock**	**18°C Bm**	**18°C Bm + Sat**	**25°C Mock**	**25°C Bm**	**25°C Bm+Sat**
Total	16,913,661	17,910,527	19,097,617	16,593,227	17,191,750	17,781,409
BBSV		12,401,345 (69.2%)	5,651,525 (29.6%)		9,683,060 (56.3%)	5,438,352 (30.6%)
Sat-RNA			10,575,344 (43.2%)			8,688,464 (30.8%)

**Figure 3:**
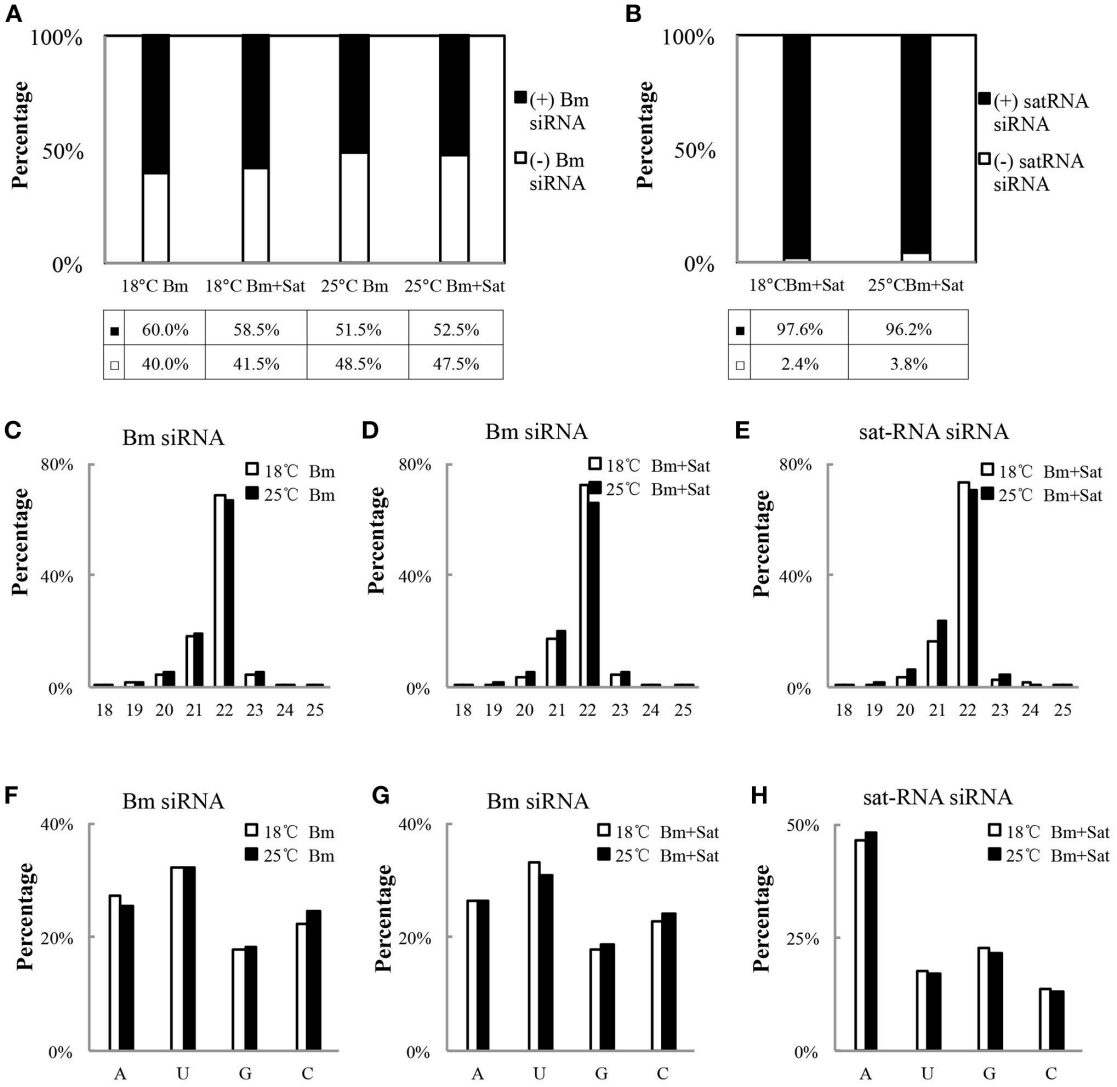
**Profiles of the Bm- and sat-RNA -derived siRNAs**. The total siRNAs were isolated from Bm-inoculated and sat-RNA co-inoculated systemic leaves grown at 18 and 25°C conditions at 12 dpi. The 18- to 30-nt siRNA from Bm- and sat-RNA were analyzed. Relative abundance of siRNA from the positive strand (black column) and the negative strand (white column) of BBSV genomic RNA **(A)** or sat-RNA **(B)**. The relative percentages of (+) siRNA and (−) siRNA to total siRNAs are shown in the bottom. **(C–E)** showed the size distributions of Bm- and sat-RNA- derived siRNAs in the different treatment as indicated. **(F–H)** showed relative frequency of 5′-terminal nucleotide of Bm- and sat-RNA -derived siRNAs with respect to the amount of individual nucleotides in the Bm genome **(F,G)** or sat-RNA genome **(H)**.

We further analyzed the polarity of virus-derived siRNAs and found different profiles of viral siRNAs derived from Bm and sat-RNA. Nearly equal amount of positive and negative stranded Bm-siRNAs accumulated in all small RNA samples (Figure 3A). However, in sat-RNA co-infected leaves, a clear prevalence for sense strand of sat-siRNAs was observed under both 18 and 25°C temperature conditions, representing 97.6 and 96.2% of the total sat-siRNAs, respectively (Figure 3B). The distinct polarity of Bm- and sat-siRNAs might due to their different replication processes, in which, positive-stranded RNA viruses usually use dsRNA as an intermediate template for genomic RNA synthesis (Kovalev et al., 2014), and most circular satellite RNAs utilize rolling cycle mechanism for its replication that produce abundant plus strands and few minus templates (Branch and Robertson, 1984; Bruening et al., 1991).

With regard to size distribution, both Bm-siRNAs and sat-siRNAs are dominated by 22-nt reads (65.3–73.6%), followed by 21-nt reads (16.3–28.9%) and other length reads (Figures 3C–E), which suggests that the *N. benthamiana* homologs of DCL2 and DCL4 are the predominant Dicers involved in the biogenesis of viral siRNA from both Bm and sat-RNA. The dominance of 22-nt siRNAs of BBSV and sat-RNA is consistent with *Cymbidium ring spot virus* (CymRSV)-derived vsRNAs in *N. benthamiana* plants (Donaire et al., 2009).

The previous studies have reported that the selective loading of small RNAs into specific AGOs is determined by the 5′-terminal nucleotides of siRNAs (Mi et al., 2008; Montgomery et al., 2008). To determine potential interactions between viral siRNAs and distinct AGO complexes, we analyzed the 5′ terminial of viral siRNAs derived from Bm- and sat-siRNAs (Figures 3F–H). Bm-siRNAs are dominated by uridines (5′U) with the ratio of 31.0–33.2%, and followed in order by adenines (A), cytidines (C), and guanines (G), which is consistent in the samples infected by Bm alone or with sat-RNA under different temperature (Figures 3F,G). In contrast, sat-RNA-derived siRNAs exhibited a clear predominance of A at 5′ end (46.2–48.0%) (Figure 3H). These trends were not affected by two temperature conditions. In contrast, there is no obvious preference of nucleotides in the composition of BBSV and sat-RNA genome (BBSV: A 24.8%, C 23.7%, G 25.8%, U 25.7%; sat-RNA: A 26.0%, C 22.0%, G 24.4%, U 27.6%). Considering different AGOs preferred different 5′-terminal first nucleotide (Mi et al., 2008; Montgomery et al., 2008), these results indicate an involvement of different AGOs, mainly AGO1, and AGO2, in the antiviral silencing targeting Bm and sat-RNA, respectively.

### Sat-RNA-derived siRNAs are predominantly processed from the highly structured region of the sat RNA genome

**Figure 4:**
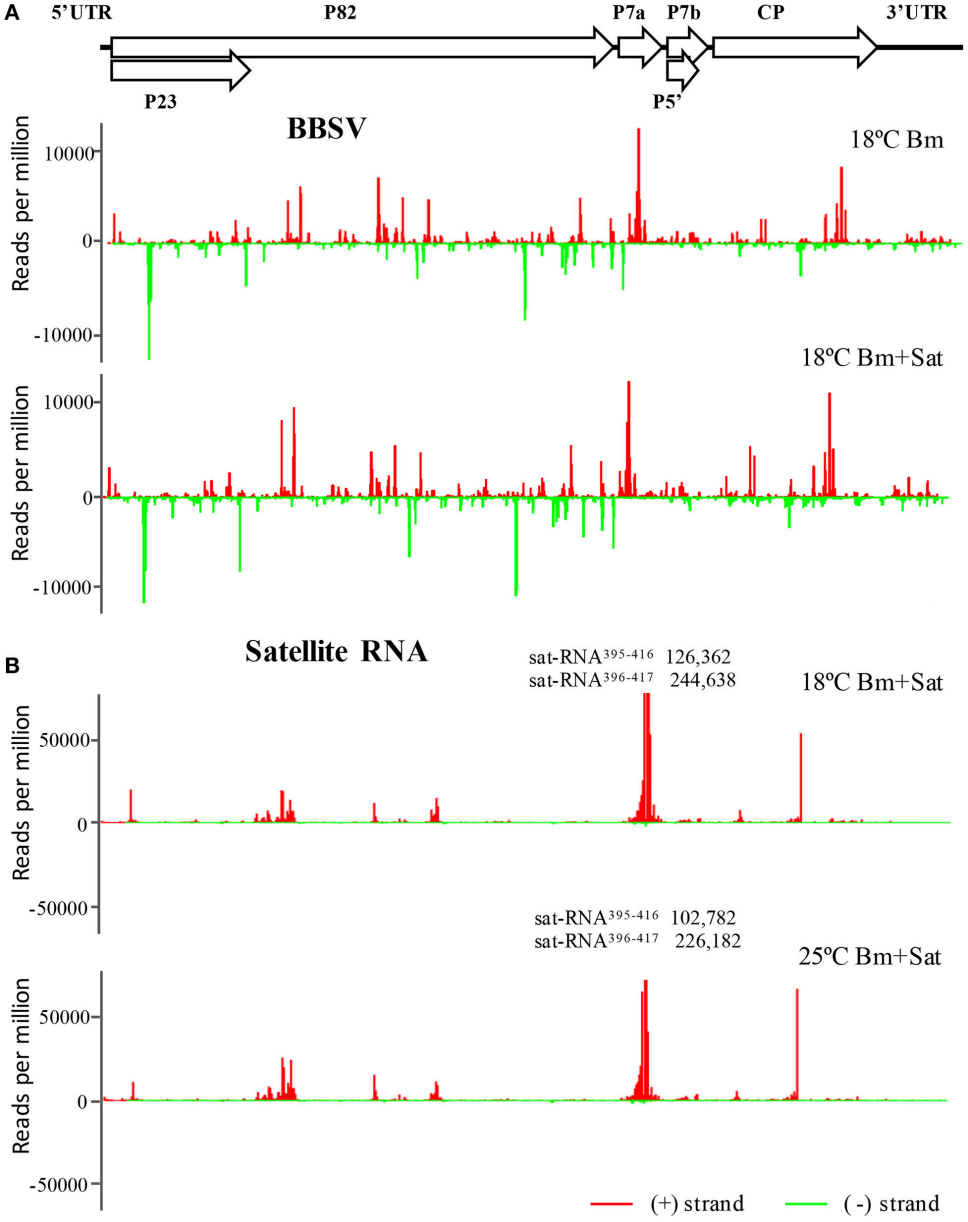
**The abundant distribution of siRNAs on Bm and sat-RNA genomes**. siRNAs derived from the viral genome of Bm **(A)** and sat-RNA **(B)** are shown in red above (positive strand) or green below (negative strand) the horizontal line. X axis represents the length of the genome, and Y axis the counts of the siRNAs per million of total viral-derived siRNAs. The Bm genome organization is shown schematically above the graphs with coding open reading frames indicated as open arrows. Note that the read counts of two (+) sat-siRNAs (sat-RNA^395−416^ and sat-RNA^396−417^) are out of scale and indicated **(B)**.

To further explore the frequencies of Bm-siRNAs and sat-siRNAs in the Bm and sat-RNA genomes, we mapped the positive- and negative-stranded viral siRNAs to the top and bottom of genomes of Bm and sat-RNA, respectively. Note that two different scales were used to accommodate the high abundance of siRNAs. The Bm-siRNAs were almost continuously but heterogeneously distributed along Bm genome and exhibited similar patterns with or without sat-RNA (Figure 4A). However, the sat-siRNAs exhibited several peak distribution features in all sat-RNA co-inoculation samples (Figure 4B). The most abundant sat-siRNAs were peaked in the positive strand of nt 396–417 (Figure 4B), where a highly structured region was predicted to be formed by using Mfold software (Figure S1). Notably, the distribution patterns of Bm- and sat-RNA-derived siRNAs remained unchanged in all the virus-inoculated samples at both 18 and 25°C, indicating that the profiles of Bm- and sat-siRNAs were not a result of sequencing biases (Figure 4, Figure S2).

### Bm infection or co-infection with Bm sat-RNA perturbs the expression of antiviral silencing genes

**Figure 5:**
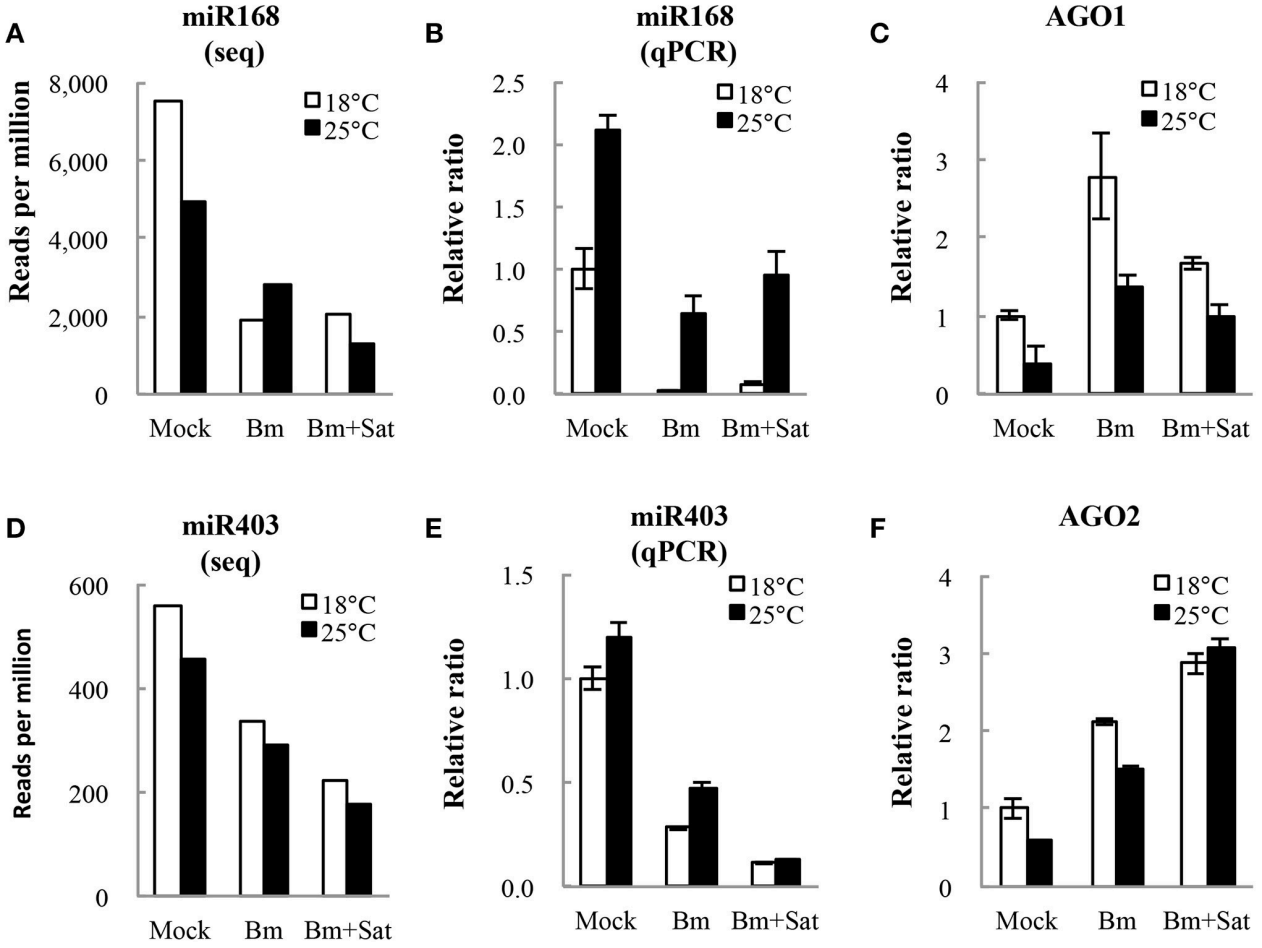
**miR168 and miR403 expression levels and their targets AGO1 and AGO2 analysis. (A,D)** showed the amount of miR168 and miR403 per million total reads in small RNA library. **(B,E)** showed quantitative RT-PCR validation of the relative expression levels of miR168 and miR403. **(C,F)** showed relative expression of AGO1 and AGO2 transcripts targeted by miR168 and miR403, respectively. Error bars represent mean standard error calculated from three biological replicates.

We also analyzed miRNA expression from total small RNA reads. Virus infection induced down-regulation of miR164, miR166, miR167, miR168, and miR403, and up-regulation of miR172 and miR397, after inoculation of Bm alone or with sat-RNA at two temperatures (Figures 5A,D; Figure S3), indicating that a series of host miRNAs were affected by virus infection. Among these miRNAs, miR168, and miR403 target the mRNAs of *AGO1* and *AGO2*, respectively, which are the main antiviral silencing components. Therefore, the expression levels of miR168 and miR403 were further confirmed by quantitative RT-PCR (Figures 5B,E). Interestingly, the down-regulation of miR168 level was more obvious in Bm samples than that in sat-RNA co-inoculation samples, which is consistent in 18 and 25°C (Figure 5B). Accordingly, *AGO1*, the target of miR168, was up-regulated and the mRNA level was higher in Bm samples than that with sat-RNA (Figure 5C), suggesting a main role of antiviral AGO1 in Bm inoculation leaves. However, miR403, which negatively regulates AGO2 mRNAs, exhibited down-regulation level to a larger extent in sat-RNA co-inoculation samples than that in Bm samples (Figure 5E), and its target *AGO2* were up-regulated higher in sat-RNA co-inoculation leaves (Figure 5F), indicating AGO2 as a major antiviral component of RNA silencing in sat-RNA involving leaves. These results were consistent with the results of 5′ nucleotide bias analysis of viral siRNAs, in which predominant U in Bm-siRNAs were mediated by AGO1, whereas prominent A preference in sat-siRNAs were mainly AGO2 involved (Figures 2A,B).

**Figure 6:**
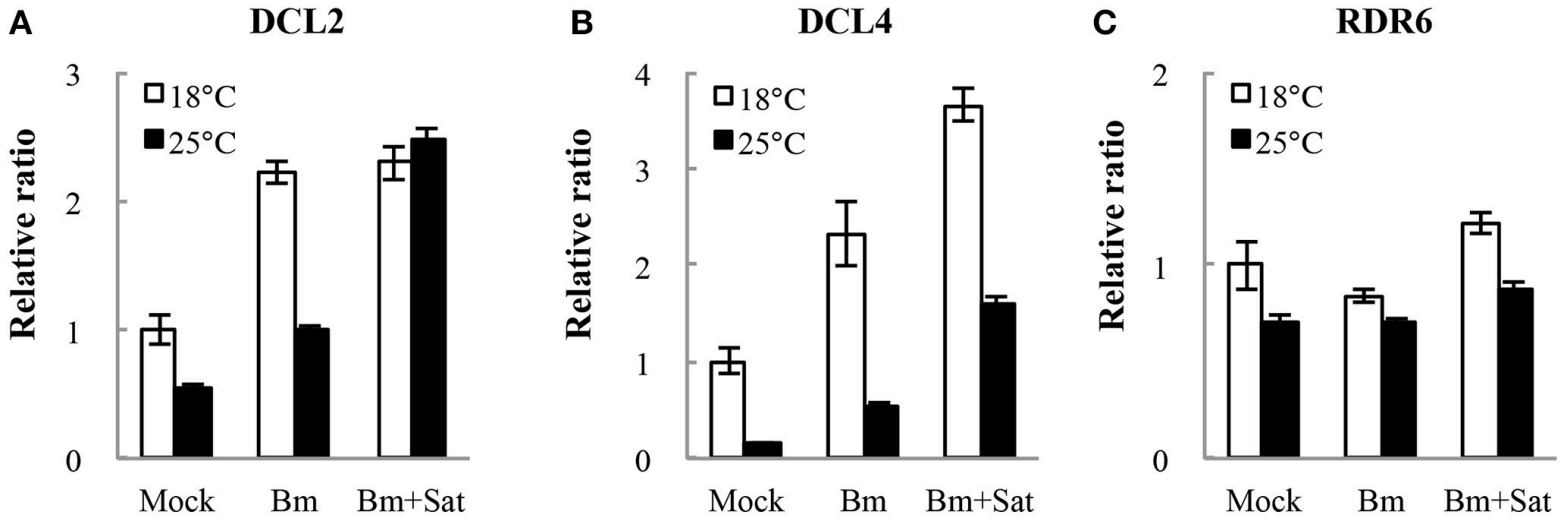
**Quantitative RT-PCR analysis of the expression of RNA silencing-related ***N. benthamiana*** DCL2 (A), DCL4 (B), and RDR6 (C) mRNAs**. Error bars represent mean standard error calculated from three biological replicates. The information and primer sequences used for amplification of DCLs and RDR6 were listed in Table S1.

We also detected the accumulation of other RNA silencing components *DCL2, DCL4*, and *RDR6* mRNA levels in *N. benthamiana* by quantitative real-time RT-PCR (Figure 6). Compared with mock, Bm alone or with sat-RNA consistently induced relatively high levels of *DCL2* and *DCL4* mRNA accumulation (Figures 6A,B), which is consistent with the sequencing data of dominant 22- and 21-nt length siRNAs. It is interesting that no significant changes in the accumulation of *RDR6* mRNAs in sat-RNA co-inoculation samples compared with Bm alone (Figure 6B), perhaps due to its primary function of stably producing ta-siRNAs that is most important for plant growth. All these data suggest that the host plants exert different expression patterns to Bm and sat-RNA for antiviral silencing.

## Discussion

Sat-RNAs are viral parasites and depend on their helper viruses for replication, encapsidation and movement in the host plants. Sat-RNAs are usually involved in the interaction between their helper viruses and plant hosts by modulating the accumulation level of helper viruses and symptom induction. In this study, we found that the sat-RNA of BBSV facilitated the systemic infection of the helper virus in *N. benthamiana*. Our results also show that BBSV is temperature sensitive and the systemic infection is enhanced at lower temperature. Analysis of siRNAs derived from the sat-RNA and the helper virus by both northern blotting and cloning/sequencing revealed that virus infection triggers high levels of RNA silencing and that sat-RNA co-replication produces highly abundant sat-siRNAs to relieve the silencing pressure for helper virus. Meanwhile, virus infection induced high levels of expression of DCLs and AGOs of RNA silencing as main antiviral elements. Our findings reveal that the helper virus (Bm) is probably mainly targeted by AGO1-associated complex, and the sat-RNA is silenced by AGO2-associated complex. These results illustrate the sat-RNA could benefit the helper virus, especially when the helper virus is confronted with strong defense of host plants.

It is known that plant RNA-directed RNA polymerases (RDRs) were involved in antiviral silencing and that high temperature enhances antiviral silencing in *N. benthamiana* because RDR6 may be inactive at the low temperature (Xie et al., 2001; Szittya et al., 2003; Qu et al., 2005). In *Arabidopsis* and *N. benthamiana*, RDR6 has been shown playing an important role in the host antiviral defense (Dalmay et al., 2000; Mourrain et al., 2000; Schwach et al., 2005). Thus, our observation that milder symptom by BBSV in *N. benthamiana* at the higher temperature might be due to the improvement of RDR6 functions, but not of its mRNA levels shown in Figure 6C. The incomplete or aborted viral RNAs produced in the process of BBSV replication would be recognized as aberrant RNAs to be converted into dsRNAs *de novo* by RDR6 or other RDRs, strengthening the silencing to degrade the viral RNAs.

Although the replication of defective interfering RNA (DI RNAs), sat-RNAs and viroids produces abundant siRNAs, these sub-viral RNAs are resistant to RNA silencing, because highly structured RNAs may be poor targets for RNA cleavages by RNA-induced silencing complex (Wang et al., 2004; Du et al., 2007; Gomez and Pallas, 2007; Itaya et al., 2007). Unlike DI RNAs, however, sat-RNAs have no sequence homology with the helper virus so that the abundant siRNAs derived from the sat-RNA are not able to enhance BBSV silencing. Therefore, we propose that the efficient sat-RNA replication yields abundant substrates for host Dicers, which compete for the silencing factors and thereby facilitate infection and spread of the helper virus. It should be pointed out that sat-RNA modulation of helper virus silencing reported in this study is independent of the sequence homology between sat-RNA and the helper virus reported previously for TCV sat-RNA C, which is 3′ co-terminal with the helper viral RNAs (Zhang and Simon, 2003).

It is known that sat-RNAs compete for the replication machinery with the helper virus, which could enhance the survival of the host to the benefit of the helper virus (Hull, 2002). Our work suggests a new function for sat-RNAs in the antiviral silencing of the helper virus that is also beneficiary to the infection and spread of the helper virus. We propose that these properties of sat-RNAs play a key role in the emergence and evolution of sat-RNAs.

## Author contributions

XW and JY conceived and designed the experiments; JX and DeL performed the experiments; JX and XW analyzed the data and drafted the manuscript. YZ, YW, CH and DaL participated in experimental coordination and revision of the manuscript; XW and JY proofread and finalized the manuscript.

## Funding

This work was supported by grants from National Science Foundation of China (31370176, 31500123, and 31322004).

### Conflict of interest statement

The authors declare that the research was conducted in the absence of any commercial or financial relationships that could be construed as a potential conflict of interest.
