# Supplementary material for: Improved Pathogenicity of a Beet Black Scorch Virus Variant by Low Temperature and Co-infection with Its Satellite RNA
Source: Front Microbiol. 2016 Nov 4;7:1771. doi: 10.3389/fmicb.2016.01771 (PMC5095503; doi:10.3389/fmicb.2016.01771)
Supplement: Supplementary file 1 [file Table1.docx]

**Table S1 Primers used in this paper**

| Primer ID | Primer Sequence (5’-3’) | Brief Description |
| --- | --- | --- |
| Oligo dT18 | TTTTTTTTTTTTTTTTT | Reverse transcription primer for EF1α, DCL2, DCL4, AGO1, AGO2, and RDR6 |
| EF1α FP | AGCTTTACCTCCCAAGTCATC | For EF1α stem-loop RT-PCR and Real time RT-PCR |
| EF2α RP | AGAACGCCTGTCAATCTTGG | For EF1α stem-loop RT-PCR and Real time RT-PCR |
| DCL2 FP | GCGTGGCGTATATTACAGTAGAG | For DCL2 Real time RT-PCR |
| DCL2 RP | TCAGATGCACATTCCTTCAGC | For DCL2 Real time RT-PCR |
| DCL4 FP | CGTCCGTGCCCAGAAATCT | For DCL4 Real time RT-PCR |
| DCL4 RP | AATGCAATTGCCGCTTTGA | For DCL4 Real time RT-PCR |
| AGO1 FP | AGATCTGTACAAGACTTGGC | For AGO1 Real time RT-PCR |
| AGO1 RP | TTATTGGCAAACAACCTAGT | For AGO1 Real time RT-PCR |
| AGO2 FP | CATTTGAACCTCCTTTCTATCGAC | For AGO2 Real time RT-PCR |
| AGO2 RP | CATACCTCTAGAAGTGAGGACCAC | For AGO2 Real time RT-PCR |
| RDR6 FP | TTCAGGAATGTCTTCGAGCG | For RDR6 Real time RT-PCR |
| RDR6 RP | AGTGATCTAGCAACCCAATGAG | For RDR6 Real time RT-PCR |
| miR168 RT ^a^ | GTTGGCTCTGGTGCAGGGTCCGAGGTATTCGCACCAGAGCCAACTTCCCGACC | For miR168 RT |
| miR403 RT ^a^ | GTTGGCTCTGGTGCAGGGTCCGAGGTATTCGCACCAGAGCCAACCGAGTTTGT | For miR403 RT |
| Universal RP | GTGCAGGGTCCGAGGT | universal reverse primer for stem loop RT-PCR |
| miR168 FP ^b^ | TGCGGTCGCTTGGTGCAG | Forward primer for miR168 Real time RT-PCR |
| miR403 FP ^b^ | CGGCGGTTAGATTCACGCA | Forward primer for miR403 Real time RT-PCR |

Note: ^a^, Reverse-complement sequence of small RNA is underlined; ^b^, small RNA sequence is underlined.
