# Supplementary figures and images for: Improved Pathogenicity of a Beet Black Scorch Virus Variant by Low Temperature and Co-infection with Its Satellite RNA
Source: Front Microbiol. 2016 Nov 4;7:1771. doi: 10.3389/fmicb.2016.01771 (PMC5095503; doi:10.3389/fmicb.2016.01771)

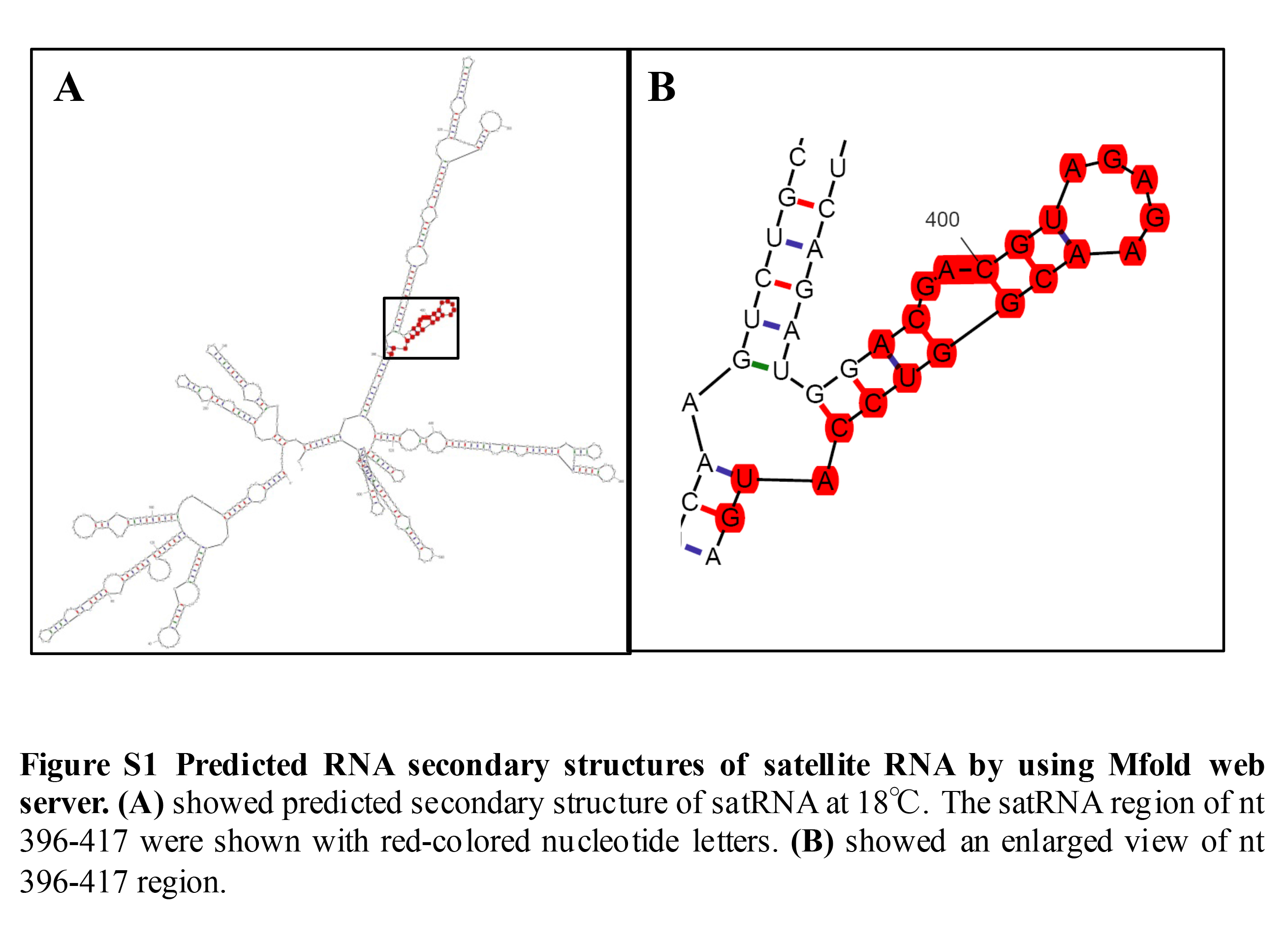

Supplement: Supplementary file 3 [file Image1.JPEG]

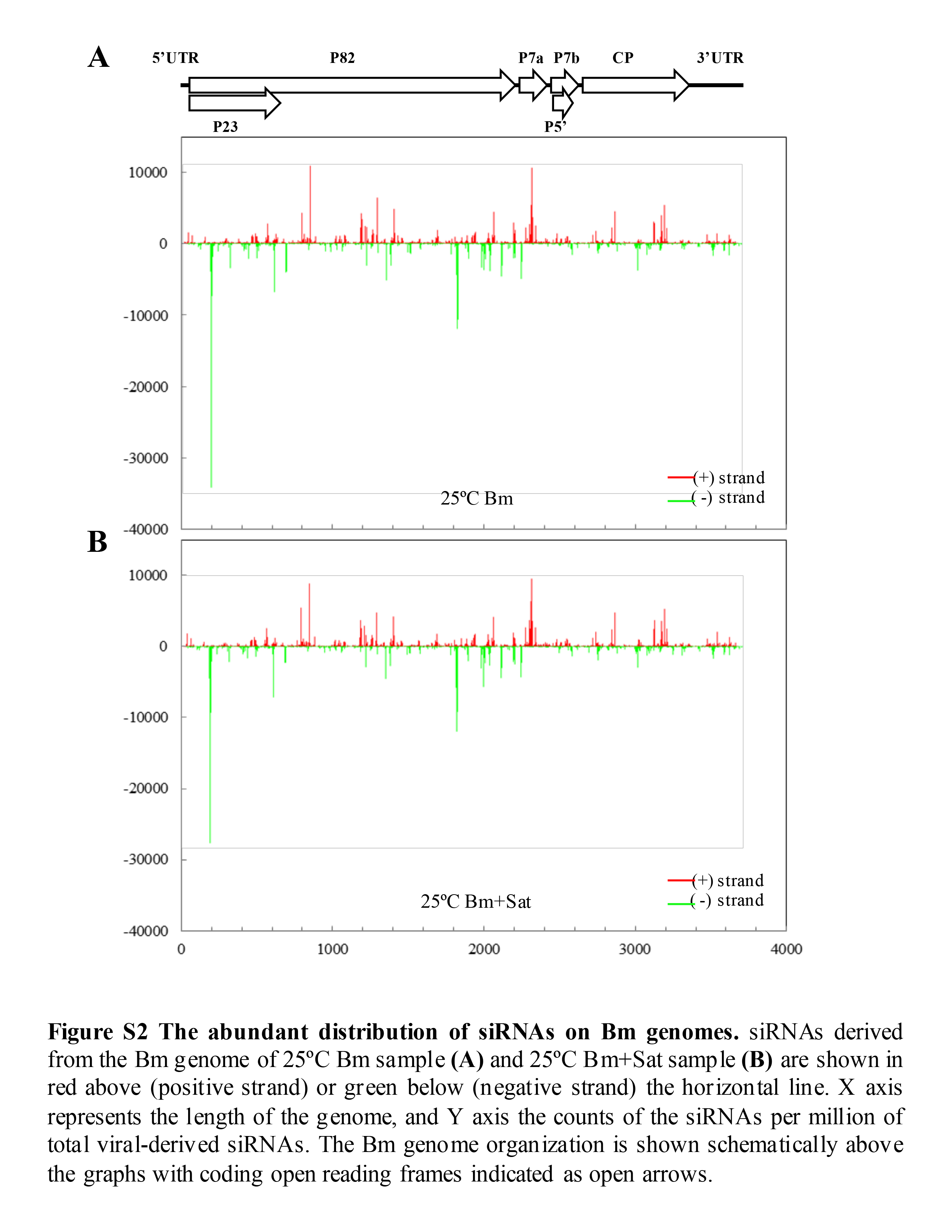

Supplement: Supplementary file 4 [file Image2.JPEG]

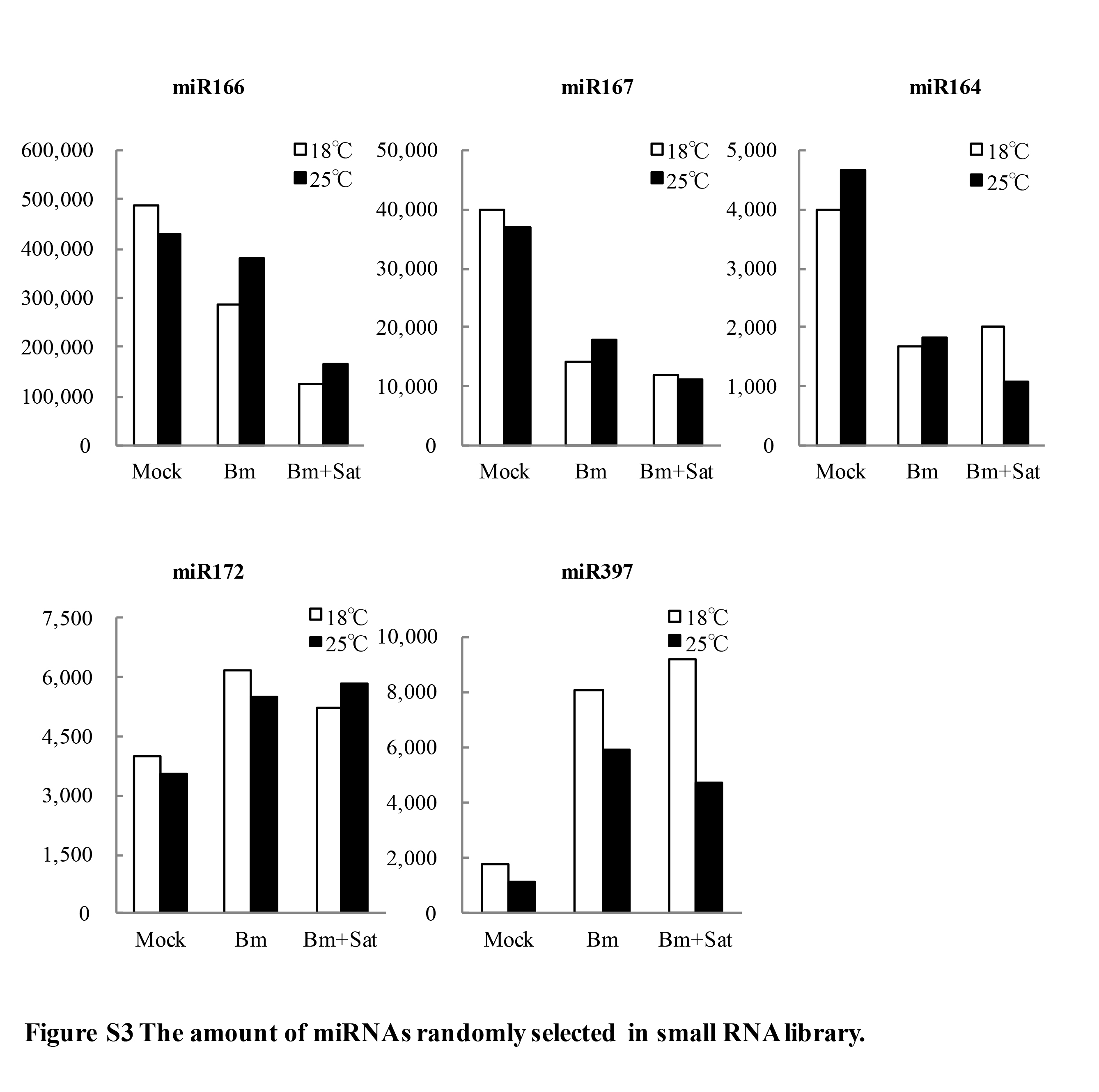

Supplement: Supplementary file 5 [file Image3.JPEG]
